# Preliminary Evidence of Sex Differences in Cortical Thickness Following Acute Mild Traumatic Brain Injury

**DOI:** 10.3389/fneur.2018.00878

**Published:** 2018-10-17

**Authors:** Meihua Shao, Jieli Cao, Lijun Bai, Wenming Huang, Shan Wang, Chuanzhu Sun, Shuoqiu Gan, Limei Ye, Bo Yin, Danbin Zhang, Chenghui Gu, Liuxun Hu, Guanghui Bai, Zhihan Yan

**Affiliations:** ^1^Radiology Department of the Second Affiliated Hospital and Yuying Children's Hospital, Wenzhou Medical University, Wenzhou, China; ^2^The Key Laboratory of Biomedical Information Engineering, Ministry of Education, Department of Biomedical Engineering, School of Life Science and Technology, Xi' an Jiaotong University, Xi' an, China; ^3^Neurosurgery Department of the Second Affiliated Hospital and Yuying Children's Hospital of Wenzhou Medical University, Wenzhou, China

**Keywords:** mild traumatic brain injury, cortical thickness, gender difference, interaction effect, clinical outcomes

## Abstract

The main objective of this study was to evaluate sex differences in cortical thickness after acute mild traumatic brain injury (mTBI) and its associations with clinical outcomes. Thirty-two patients with mTBI at acute phase (2.4 ± 1.3 days post-injury) and 30 healthy controls were enrolled. All the participants underwent comprehensive neurocognitive assessments and MRI to assess cortical thickness. Significant sex differences were determined by using variance analysis of factorial design. Relations between the cortical thickness and clinical assessments were measured with the Spearman Correlation. Results revealed that patients with mTBI had significantly reduced cortical thickness in the left entorhinal cortex while increased cortical thickness in the left precuneus cortex and right lateral occipital cortex, compared with healthy controls. The interaction effect of the group × sex on cortical thickness was significant. Female patients had significant thicker cortical thickness in the left caudal anterior cingulate cortex (ACC) than male patients and had higher scores on Posttraumatic stress disorder Checklist—Civilian Version (PCL-C). Spearman correlational analysis showed a significantly positive correlations between the cortical thickness of the left caudal ACC and PCL-C ratings in female patients. Sex differences in cortical thickness support its potential as a neuroimaging phenotype for investigating the differences in clinical profiles of mild TBI between women and men.

## Introduction

Traumatic brain injury (TBI) is an important global health issue, of which 75–90% are classified as mild TBI (mTBI) (1). Although most patients with mTBI become asymptomatic within days to weeks, some develop a series of persistent symptoms that have been called as “persistent post-concussive syndrome.” There are several factors associated with those various outcomes, but one of the most controversial and interesting factors is sex (2). Sex differences in outcomes after mTBI have been addressed in lots of studies, of which some found females have a poorer outcome than males (3–8). Another study have found that females have a higher risk of developing epilepsy, suicide, and use more health care services and males have a higher risk for schizophrenia after mTBI (9). While other studies have shown no substantial difference in outcome with regard to sex (10, 11). The effect of sex on outcome after mTBI is less clear. Observation studies may be confounded by many factors, including different symptoms reporting among women and men (12, 7). Therefore, a more objective measurement, such as imaging findings, is crucial in avoiding such bias. Understanding sex differences of brain injury mechanism after mild TBI may change the future diagnostic work-up in patients with mTBI and lead to separate management strategies for patients of different sex.

Sex differences in brain activities after TBI have been reported in recent years. An fMRI study in veterans with TBI reveals that males showed increased functional connectivity between the left orbitofrontal cortex (OFC) and the right mid frontal cortex compared with females. In the meantime, a significant negative association is found on the overall score on the Buss Perry Aggression Questionnaire with functional connectivity between the left OFC and left angular region in male veterans (13). Diffusion tensor imaging (DTI) shows that male patients with mTBI have decreased fractional anisotropy (FA) in the uncinate fasciculus (UF) compared with female patients and was negatively correlated with time to symptom resolution (14). However, there is lack of findings on the cortical thickness alternations following mild TBI. Cortical thickness is an intrinsic biological parameter and should be independent of external factors such as the MRI scanner type, imaging sequence, spatial resolution and/or field strength (15, 16). Sex differences in cortical thickness have been well-documented in healthy adults (17). Cortical thickness alternations are also reported in the chronic phase of various TBI (from vary mild, moderate to more serve TBI) (18–20), there is no study focused on the sex differences in cortical thickness following acute mTBI. Moreover, gender difference in outcomes, favoring females as endogenous neuroprotectants, has been documented in TBI. However, a consistent finding in the research literature on general traumatic experience is that women exhibit twice the rate of the disorder as men, in spite of men experiencing greater lifetime exposure to traumatic events overall (21–23). We hypothesized that there was a significant interaction effect of gender and diagnosis in the traumatic complaints and associated cortical thickness alternations following mTBI.

## Materials and methods

### Participants

All consecutive patients with non-contrast head CT due to acute head trauma enrolling from the local emergency department (ED) formed the initial population. Inclusion criteria for all mTBI patients were based on the World Health Organization's Collaborating Center for Neurotrauma Task Force (24): (i) Glasgow Coma Scale (GCS) score of 13–15, (ii) one or more/any of the following: loss of consciousness (LOC) for < 30 min, posttraumatic amnesia (PTA) for 24 or less hours, and/or other transient neurological abnormalities such as focal signs, seizure, and intracranial lesions not requiring surgery, (iii) within 1 week after onset of a mTBI, (iv) were aged 16 years or older. Mild TBI patients were excluded for: (1) a history of a previous brain injury, neurological disease, long-standing psychiatric condition, concurrent substance, or alcohol abuse, (2) a structural abnormality on neuroimaging (CT and MRI), (3) intubation and/or presence of a skull fracture and administration of sedatives, (4) the manifestation of mTBI due to medications by other injuries (e.g., systemic injuries, facial injuries, or spinal cord injury), (5) other problems (e.g., psychological trauma, language barrier, or coexisting medical conditions), (6) caused by penetrating craniocerebral injury.

Thirty-two patients with mTBI (18 males) and 30 sex-, age-, and education-matched healthy controls (14 males) without neurologic impairment or psychiatric disorders participated in the study. Participants were all right-handed according to the Edinburgh Handedness Inventory (25). All the subjects gave written, informed consent in person approved by a local institutional review board and conducted in accordance with the Declaration of Helsinki.

### Image acquisition

A non-contrast CT scan was performed on all consecutive patients following acute head injury with a 64-row CT scanner (GE, Lightspeed VCT). All the patients with mTBI went through the MRI scans the day they were recruited in the group. The MRI scans were acquired with the use of 3T MRI scanner (GE 750). A custom-built head holder was used to prevent head movements. The MRI protocol involved the high-resolution T1-weighted 3D BRAVO sequence (echo time = 3.4 ms, repetition time = 7.7 ms, flip angle = 9°, slice thickness = 1 mm, field of view = 256 × 256 mm, matrix size = 256 × 256). The presence of focal lesions and cerebral microbleeds was independently determined by experienced clinical neuroradiologists (with 9 and 10 years' experience) who assessed multiple modalities of neuroimaging data acquired at baseline (T1-flair, T2-flair, T2, susceptibility weighted imaging). Any disagreement between these two observers was resolved by consensus. None of patients were with visible contusion lesions using conventional neuroimaging techniques or exhibited cerebral micro-bleeds on SWI.

### Clinical assessments

Clinical assessments were performed within 48 h of MR imaging for all the participants. Based on these previous publications, a limited set of neuropsychological tests were analyzed in the current study, to reduce multiple testing issues. This selection was based on our previous work demonstrating sensitivity to TBI-related alterations to brain structure (26, 27). The following tests were assessed: (i) Trail-Making Test Part A and Digit Symbol coding score from the Wechsler Adult Intelligence Scale III (WAIS-III) to examine cognitive information processing speed (28); (ii) Forward Digit Span and Backward Digit Span from the WAIS-III to assess immediate auditory span, working memory, and executive function (29); (iii) Verbal Fluency Test to assess verbal fluency including language ability, semantic memory and executive function (30); (iv) Posttraumatic stress disorder Checklist—Civilian Version (31). In addition, post concussive symptoms (PCS) were measured with the Rivermead Post-Concussion Symptom Questionnaire (RPCS) (32).

### Cortical thickness analyses

We used FreeSurfer version 5.3.0 (33) (https://surfer.nmr.mgh.harvard.edu/fswiki) to extract surface-based features from the high-resolution T1-weighted images. The reliability of obtaining cortical thickness measurements from MRI scans has been well validated (15, 34, 35). The high- resolution T1-weighted MR volume for each participant was bias corrected, skull stripped, and segmented into white matter, gray matter, and cerebrospinal fluid before surface-based morphometry (15). Then, we conducted to tessellate the gray-white boundary, perform automated topology correction, and perform surface deformation to locate the gray-white and gray-pial boundaries (36, 37). Cortical thickness was calculated as the closet distance between the gray-white matter boundary and the pial mesh at each vertex on the tessellated surface (37). The surface was then anatomically parcellated by using the Desikan-Killiany atlas into 66 structures (33 structures for each hemisphere) (38). Accuracy for automated processing was inspected by an expert (with 4 years of experience in editing data from more than 200 examinations and trained in this field) and manual corrections were applied if necessary.

We mapped these structures onto a spherical space to achieve point-to-point correspondence for each subject (39). The final segmentation of surface-based labeling was based on both a subject-independent probabilistic atlas and on subject-specific measured values. Combining the cortical-thickness map and surface-based labels, we computed the average cortical thickness for each region.

### Statistical analysis

The Shapiro–Wilk *W*-test was used to test for normality distribution of all continuous variables. The independent two-sample *t-*test and the Mann–Whitney test were used to compare group differences based on data normality, respectively. Chi-square analyses were applied to assess categorical variables. *P* < 0.05 were considered to indicate a significant difference. Effect sizes (Cohen's d) were computed to demonstrate the magnitude of observed differences. Two-sample *t*-tests were used to explore cortical thickness differences between groups from each native-surface region of interest (ROIs), and results were assessed for significance after controlling the false-discovery rate (FDR) at < 0.05 to correct for multiple comparisons. The 2 × 2 (Group × Sex) mixed measures ANOVAs were performed to test the interactions and group effects, respectively. *P* < 0.05 were considered to indicate a significant difference. Simple effect was restricted to the ROIs showing significant interaction effect of group and sex. All regional results were Bonferroni-corrected by a factor of number of ROIs (N) showing significant interaction effect, corresponding to a corrected α of *P* < 0.05/N after controlling for age and education level. Spearman's correlations were conducted between clinical assessments and the region-of-interest variables, since the data were not normally distributed.

## Results

### Demographic and clinical characteristics

Thirty-seven patients with mTBI participated in this study. Of which, data from five patients were excluded because of poor MR imaging quality (*n* = 3), and excessive head motions (*n* = 2). Finally, 32 patients (18 male) were included. Thirty matched healthy controls (14 male) were also recruited. No significant difference was showed between patients with mTBI and healthy controls regarding age and education level. The average age was 33.4 years (range 14–54 years) in healthy controls, and was 31.0 years (range 13–59 years) in patients with mTBI [*F*_(1, 60)_ = 0.62, *P* = 0.43, Cohen' s *d* = −0.20]. The average education level was 11.8 years (range 1–18 years) in healthy controls, and was 9.6 years (range 1–16 years) in patients with mTBI [*F*_(1, 60)_ = 2.88, *P* = 0.1, Cohen' s *d* = −0.43]. No significant difference was in sex (χ^2^ = 0.57, *P* = 0.45). No significant difference was found in age and education level among the four groups (i.e., male and female controls and male and female patients with mTBI) [for age: *F*_(3, 58)_ = 1.98, *P* = 0.13; for education level: *F*_(3, 58)_ = 1.32, *P* = 0.27]. A detailed demographic data and clinical characteristics were summarized in the Tables 1, 2. The major mechanism of trauma was a motor vehicle accident (11 of 18 male patients [61%], 10 of 14 female patients [71%]), followed by an assault (two of 18 male patients [11%], two of 14 female patients [14%]) (*P* = 0.4).

**Table 1:** Demographic and Clinical assessments in patients of mTBI and healthy controls.

	**Patients of mTBI**				**Healthy control subjects**			
**Demographic characterstics**	**Female (*n* = 14)**	**Male (*n* = 18)**	***P***	**Cohen's d**	**Female (*n* = 16)**	**Male (*n* = 14)**	***P***	**Cohen's d**
Age	33.1 ± 14.3 (13~59)	29.3 ± 10.3 (16~48)	0.41	0.3	37.5 ± 12.3 (20~54)	28.6 ± 8.4 (14~53)	0.19	0.8
Education level	9.5 ± 4.5 (1~16)	9.7 ± 3.2 (5~15)	0.87	0.05	10.7 ± 4.7 (3~18)	12.4 ± 4.8 (1~18)	0.34	0.4
**MECHANISM OF INJURY**								
Motor vehicle accident	10 (71.4%)	11 (61.1%)						
Assault	2 (14.2%)	2 (11.1%)						
Fall	1 (7%)	2 (11.1%)						
Other	1 (7%)	3 (16.7%)						
**CLINICAL ASSESSMENTS**								
Trail making test A	55.1 ± 34.9	47.6 ± 22.5	0.712	0.3	49.4 ± 24.1	32.8 ± 22.4	0.093	0.7
RPCS	13.9 ± 8.7	6.9 ± 6.0	0.001	0.9	2.8 ± 2.8	1.1 ± 1.9	0.391	0.7
PCL-C	26.4 ± 8.6	21.0 ± 2.9	0.001	0.8	17.0 ± 0.0	17.0 ±0.0	1.000	Non
DSC	37.4 ± 17.0	35.2 ± 14.3	0.685	0.1	42.8 ± 17.0	54.7 ± 11.8	0.035	−0.8
Forward DS	8.2 ± 1.9	8.0 ± 1.5	0.705	0.1	7.7 ± 1.7	9.1 ± 1.1	0.020	−0.9
Backward DS	3.6 ± 1.7	4.3 ± 1.8	0.247	−0.39	3.8 ± 0.9	5.7 ± 2.0	0.002	−1.3
VF	17.2 ± 5.0	17.1 ± 6.0	0.931	0.01	17.2 ± 5.7	21.3 ± 5.7	0.052	−0.7

*RPCS, Rivermead Post-Concussion Symptom Questionnaire; PCL-C, Posttraumatic stress disorder Checklist—Civilian Version; DSC, Digit Symbol coding; DS, Digit Span; VF, Verbal Fluency. Effect sizes reported are Cohen's d-values*.

**Table 2:** Demographic and Clinical assessments in female participants and male participants.

	**Female participants**				**Male participants**	
**Characteristic**	**MTBI patients**	**Healthy control**	***P***	**Cohen's d**	**MTBI patients**	**Healthy control**	***P***	**Cohen's d**
Age	33.1 ± 14.3 (13~59)	37.5 ± 12.3 (20~54)	0.18	−0.3	29.3 ± 10.3 (16~48)	28.6 ± 8.4 (14~53)	0.93	0.07
Education level	9.5 ± 4.5 (1~16)	10.7 ± 4.7 (3~18)	0.49	−0.26	9.7 ± 3.2 (5~15)	12.4 ± 4.8 (1~18)	0.07	−0.7
**CLINICAL ASSESSMENTS**								
TMT-A	55.1 ± 34.9	49.4 ± 24.1	0.862	0.2	47.6 ± 22.5	32.8 ± 22.4	0.124	0.7
RPCS	13.9 ± 8.7	2.8 ± 2.8	0.000	1.7	6.9 ± 6.0	1.1 ± 1.9	0.004	1.3
PCL-C	26.4 ± 8.6	17.0 ± 0.0	0.000	1.5	21.0 ± 2.9	17.0 ± 0.0	0.012	1.9
DSC	37.4 ± 17.0	42.8 ± 17.0	0.342	−0.3	35.2 ± 14.3	54.7 ± 11.8	0.001	−1.5
Forward DS	8.2 ± 1.9	7.7 ± 1.7	0.366	0.3'	8.0 ± 1.5	9.1 ± 1.1	0.062	−0.8
Backward DS	3.6 ± 1.7	3.8 ± 0.9	0.860	−0.1	4.3 ± 1.8	5.7 ± 2.0	0.023	−0.7
VF	17.3 ± 5.0	17.2 ± 5.7	0.962	0.01	17.1 ± 6.0	21.3 ± 5.7	0.042	−0.7

*TMT-A, Trail making test A; RPCS, Rivermead Post-Concussion Symptom Questionnaire; PCL-C, Posttraumatic stress disorder Checklist—Civilian Version; DSC, Digit Symbol coding; DS, Digit Span; VF, Verbal Fluency. Effect sizes reported are Cohen's d-values*.

ANCOVAs (Group × Sex) analysis on the clinical assessments were conducted. The interaction effect of the group × sex was significant for the PCL-C [*F*_(1, 58)_ = 5.99, *P* = 0.017] with simple effect testing suggested that females presented more complaints in the PCL-C compared with male counterparts only in the patient group but not in the control group (*P* = 0.001 after Bonferroni-correction) (Table 2). For patients, more complaints on the PCL-C was presented in both female (*P* < 0.001) and male (*P* = 0.012) compared with their corresponding controls.

### Cortical thickness results

**Figure 1:**
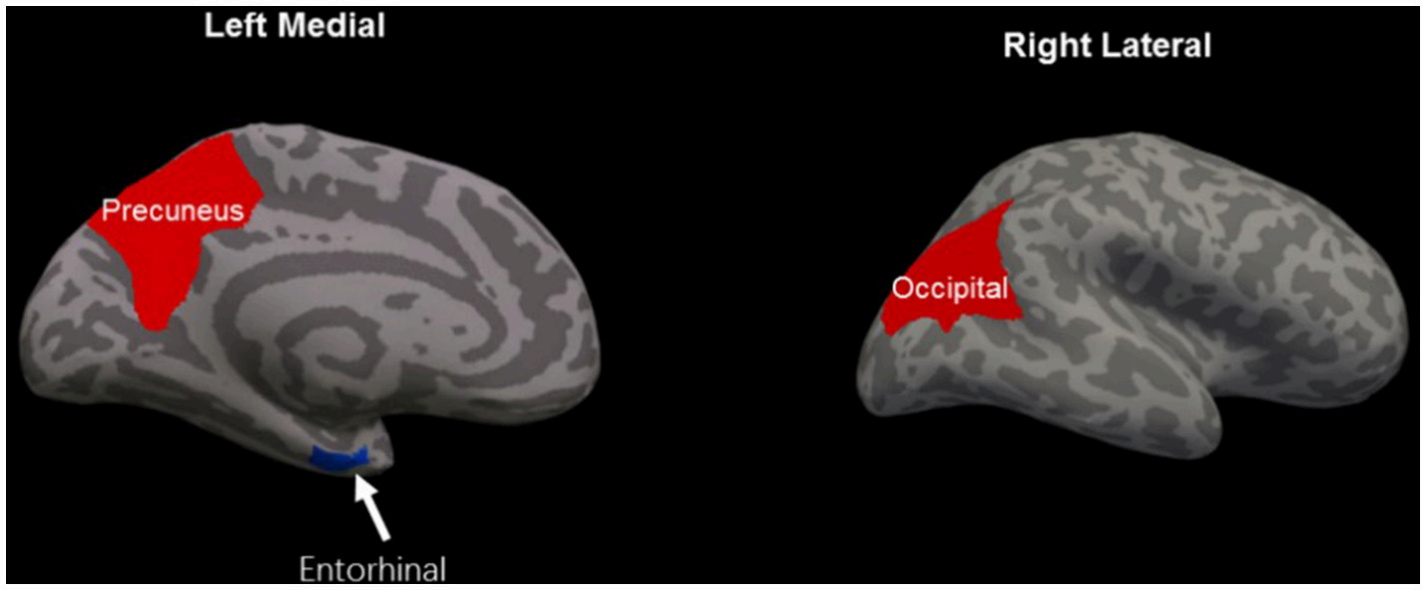
Regions of significant cortical thinning (blue) and thickening (red) in mild traumatic brain injury patients, compared with healthy controls. Regions in the left entorhinal cortex, left precuneus cortex, and right lateral occipital cortex were significant at *P* < 0.05 after FDR correction.

**Figure 2:**
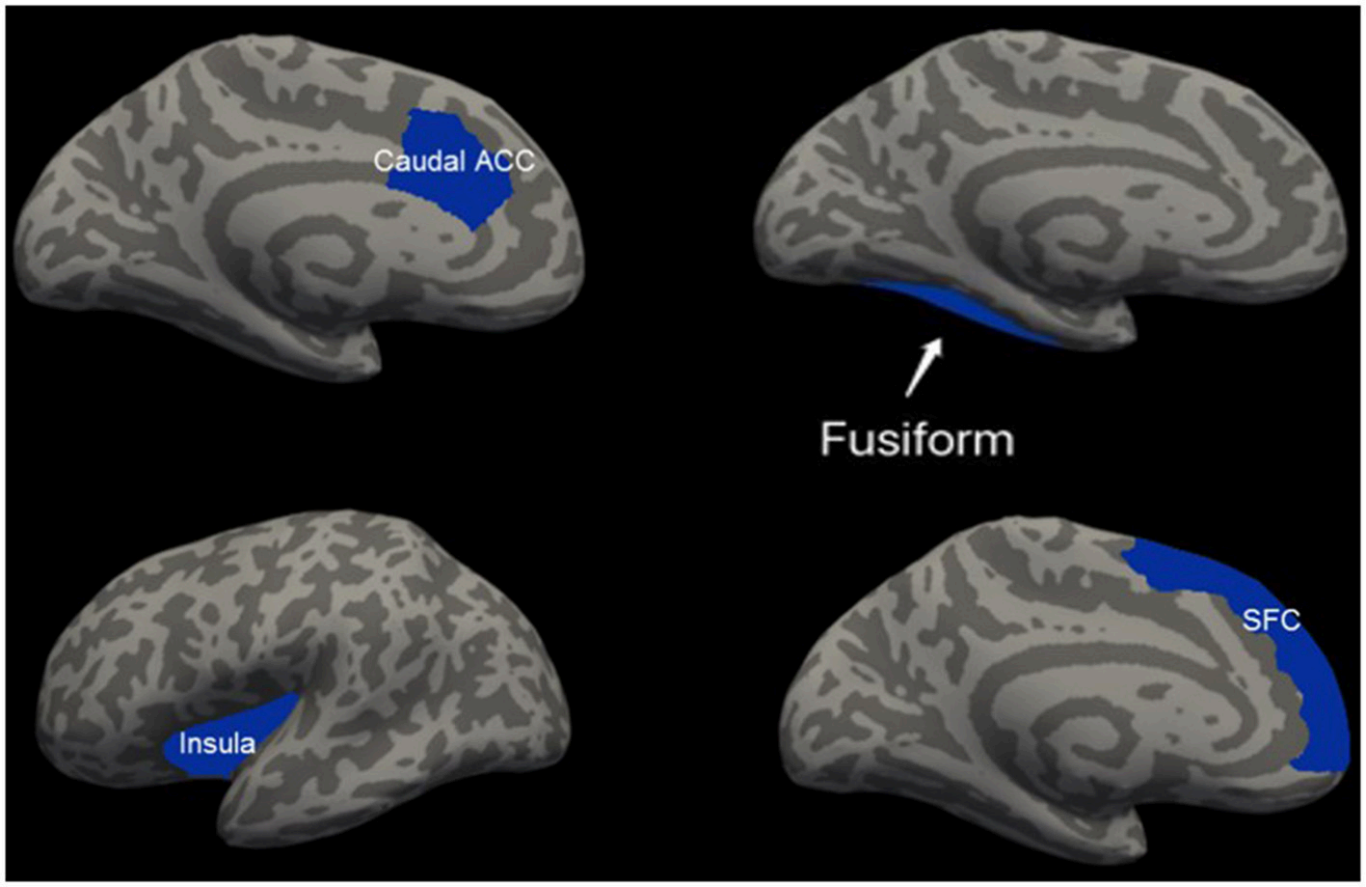
Regions of significant increased cortical thickness (blue) in female participants, compared with male participants. Left column represents in patients with mTBI and right column represents in healthy controls. Regions in the caudal anterior cingulate cortex (caudal ACC), fusiform cortex and superior frontal cortex (SFC) in the left hemisphere were significant at *P* < 0.0125 after Bonferroni-correction. Region in the left insula cortex was not significant at *P* = 0.036 after Bonferroni-correction.

Patients with mTBI presented prominently reduced cortical thickness than healthy controls in the left entorhinal cortex, while significantly increased cortical thickness in the left precuneus cortex and right lateral occipital cortex (*P* < 0.05, FDR corrected, Figure 1). The interaction effect of the group × sex on cortical thickness was significant in the left caudal anterior cingulate cortex (caudal ACC) [*F*_(1, 57)_ = 5.976, *P* = 0.018], fusiform cortex [*F*_(1, 57)_ = 10.13, *P* = 0.002], insula cortex [*F*_(1, 57)_ = 7.35, *P* = 0.009] and superior frontal cortex (SFC) [*F*_(1, 57)_ = 5.131, *P* = 0.027] (Figure 2). Simple effect testing indicated that female patients had significant increased cortical thickness than male patients in the left caudal ACC (*P* = 0.004). Increased cortical thickness in the left SFC and fusiform cortex (all for *P* = 0.005) were also presented in female controls compared with male controls. Female patients had non-significant tendency of increased cortical thickness than male patients in the insula cortex (*P* = 0.036). Other comparison did not obtain the significance.

### Correlation analysis results

Spearman correlation analysis was only restricted into clinical assessment (PCL-C) and regional cortical variable showing a significant interaction effect. There was a significantly positive correlations between the cortical thickness of the left caudal ACC and PCL-C ratings only in female patients (*r* = 0.594, *P* = 0.011). No other correlation was presented in either female, male patients or whole patient group.

## Discussion

To our knowledge, this is the first study to examine sex differences in cortical thickness during the very early acute post-injury of mTBI. The present study indicated a salient modulatory effect of sex on both self-reported symptomatology (PCL-C) and regional cortical thickness following acute mTBI. Female patients had significant increased cortical thickness than male patients in the left caudal ACC. The increased cortical thickness in the left caudal ACC was positively related with more complaints in the PCL-C ratings only in female patients. These findings may provide the clue to the management strategies for mTBI patients of different sexes.

Previous studies about sex differences in outcomes of mTBI revealed confounded results (4–6, 11, 13). A prior research suggested that it may be easier to admit their concussion symptoms after TBI for female athletes than male (12). Similarly, male concussion athletes presented more difficult to have a willing to report their symptoms than female athletes because of social norms (40) and the pressure to return to sports (41). Outcome differences in patients with mTBI could be masked by the subjective assessments. Our study avoids it by evaluating an objective measurement of the underlying neuroimaging detected injury using brain cortical thickness. To date, sex differences in cortical thickness have been reported in healthy individuals in several researches though no such findings were reported in mTBI (41). We found that the female healthy controls had significant increased cortical thickness than male controls in the left fusiform cortex, while this difference disappears modulated by the mild TBI (42). In addition, interaction effect of group and sex in the cortical thickness were primarily located in the four regions, including the left caudal ACC, fusiform cortex, insula cortex, and SFC. Based on these findings, sex difference in cortical thickness may be modulated by the injury.

We observed that the cortical thickness of female patients in the left caudal anterior cingulate cortex was positively related with the PCL-C scores. Previous work has shown that caudal anterior cingulate cortex is involved in motor control (43). For instance, it was reported that post-traumatic stress disorder (PTSD) is an anxiety disorder associated with the anterior cingulate cortex (ACC) (44). Results exhibited that decreased functional connectivity was observed between the caudal ACC and the precentral gyrus in veterans with PTSD compared to healthy controls. So we supposed that the thickened cortical thickness in left caudal anterior cingulate cortex may affect functional connectivity in female survivors. This possibility needs to be tested by future study to focus on dynamic structural and functional changes after mTBI. We have also found non-significant trend of thicker thickness in the left insula in the female patients, compared with male patients. Simple effect testing suggested that female patients had significant increased cortical thickness in this region than female controls that didn't found in the male groups (*P* = 0.016) after Bonferroni-correction. Such finding may be limited by the relatively small sample sizes. So we still speculated that it may due to the significant increased cortical thickness in female patients than male patients, compared with respective controls.

We did not explore the mechanisms that may contribute to cortical thickening in the current study, but several possibilities in this context can be considered. In animal studies, regional micro-edema has been found in thickened cortical regions within a day after cortical impact (45). Gray matter changes were in the form of increased, not decreased cortical thickness, which may have resulted from neuroinflammatory or other trophic process related to endocrine changes or functional compensation (46). Acute cerebral inflammatory reactions have been found to recover within months after injury and animal studies revealed thickened cortical regions became thinning over days with reduction of micro-edema (45). Unfortunately, we still need to conform such changes in cortical thickness after mTBI in a more chronical follow-up.

There are several limitations to our study. Post-injury time may be not long enough to observe cortical changing at acute phase and longitudinal analysis needs to be involved using following-up data. Furthermore, we did not evaluate the heterogeneity of injury, future studies should use additional outcome measures, including diffusion tensor imaging for structure integrity, resting state functional connectivity study for dynamic changes in functional networks, perfusion of cerebral blood flow (CBF) for brain metabolism using Arterial Spin Labeling Technology (ASL), which may be helpful in understanding the underlying pathophysiology and causes of sex differences in mTBI. Considering the selection of control may influence the detected injury pattern following mTBI, further study needs to enroll both orthopedically-injured patients and healthy subjects as different control group for comparison.

## Conclusions

In conclusion, the study presented the abnormal cortical thickness changes related to sex in patients with mTBI, which correlated with the more possibility to develop PTSD and impairments in the information processing speed. Thus, our results indicated a role for cortical thickness as a metric for evaluating the sex difference of mTBI injuries and may predicting subsequent clinical outcome.

## Author contributions

MS, LB, ZY, BY, and GB contributed to the conception of the study. MS, JC, and LB contributed significantly to analysis and manuscript preparation. MS, JC, LB, and SW performed the data analyses and wrote the manuscript. WH, SW, CS, SG, LY, DZ, CG, and LH helped perform the analysis with constructive discussions.

### Conflict of interest statement

The authors declare that the research was conducted in the absence of any commercial or financial relationships that could be construed as a potential conflict of interest.

## References

[1] Mondello S Schmid K Berger RP Kobeissy F Italiano D Jeromin A. The challenge of mild traumatic brain injury: role of biochemical markers in diagnosis of brain damage. Med Res Rev. (2014) 34:503–31. 10.1002/med.21295 23813922

[2] Laker SR Epidemiology of concussion and mild traumatic brain injury. PM R (2011) 3(10 Suppl. 2), S354–8. 10.1016/j.pmrj.2011.07.017 22035677

[3] Kraus JF Peek-Asa C McArthur D. The independent effect of gender on outcomes following traumatic brain injury: a preliminary investigation. Neurosurg Focus (2000) 8:e5. 10.3171/foc.2000.8.1.156 16906701

[4] Broshek DK Kaushik T Freeman JR Erlanger D Webbe F Barth JT. Sex differences in outcome following sports-related concussion. J Neurosurg. (2005) 102:856–63. 10.3171/jns.2005.102.5.0856 15926710

[5] Preiss-Farzanegan SJ Chapman B Wong TM Wu J Bazarian JJ. The relationship between gender and postconcussion symptoms after sport-related mild traumatic brain injury. PmR (2009) 1:245–53. 10.1016/j.pmrj.2009.01.011 19627902 PMC5237580

[6] Bazarian JJ Blyth B Mookerjee S He H McDermott MP. Sex differences in outcome after mild traumatic brain injury. J Neurotrauma (2010) 27:527–39. 10.1089/neu.2009.1068 19938945 PMC2867588

[7] Covassin T Bay E. Are there gender differences in cognitive function, chronic stress, and neurobehavioral symptoms after mild-to-moderate traumatic brain injury? J Neurosci Nurs. (2012) 44:124–33. 10.1097/JNN.0b013e318252737d 22555349

[8] Hsu HL Chen DY Tseng YC Kuo YS Huang YL Chiu WT. Sex differences in working memory after mild traumatic brain injury: a functional MR imaging study. Radiology (2015) 276:828–35. 10.1148/radiol.2015142549 25919663

[9] Cancelliere C Donovan J Cassidy JD. Is sex an indicator of prognosis after mild traumatic brain injury: a systematic analysis of the findings of the world health organization collaborating centre task force on mild traumatic brain injury and the international collaboration on mild traumatic brain injury prognosis. Arch Phys Med Rehabil. (2016) 97(2 Suppl.)**:**S5–18. 10.1016/j.apmr.2014.11.028 25666784

[10] Cantu RC Guskiewicz K Register-Mihalik JK. A retrospective clinical analysis of moderate to severe athletic concussions. PM R (2010) 2:1088–93. 10.1016/j.pmrj.2010.07.483 21145520

[11] Frommer LJ Gurka KK Cross KM Ingersoll CD Comstock RD Saliba SA. Sex differences in concussion symptoms of high school athletes. J Athl Train (2011) 46:76–84. 10.4085/1062-6050-46.1.76 21214354 PMC3017493

[12] Farace E Alves WM. Do women fare worse? A metaanalysis of gender differences in outcome after traumatic brain injury. Neurosurg Focus (2000) 8:e6. 10.3171/foc.2000.8.1.152 16924776

[13] McGlade E Rogowska J Yurgelun-Todd D. Sex differences in orbitofrontal connectivity in male and female veterans with TBI. Brain Imaging Behav. (2015) 9:535–49. 10.1007/s11682-015-9379-3 25864195 PMC4575683

[14] Fakhran S Yaeger K Collins M Alhilali L. Sex differences in white matter abnormalities after mild traumatic brain injury: localization and correlation with outcome. Radiology (2014) 272:815–23. 10.1148/radiol.14132512 24802388

[15] Han X Jovicich J Salat D van der Kouwe A Quinn B Czanner S. Reliability of MRI-derived measurements of human cerebral cortical thickness: the effects of field strength, scanner upgrade and manufacturer. Neuroimage (2006) 32:180–94. 10.1016/j.neuroimage.2006.02.051 16651008

[16] Wonderlick JS Ziegler DA Hosseini-Varnamkhasti P Locascio JJ Bakkour A van der Kouwe A. Reliability of MRI-derived cortical and subcortical morphometric measures: effects of pulse sequence, voxel geometry, and parallel imaging. Neuroimage (2009) 44:1324–33. 10.1016/j.neuroimage.2008.10.037 19038349 PMC2739882

[17] Sowell ER Peterson BS Kan E Woods RP Yoshii J Bansal R. Sex differences in cortical thickness mapped in 176 healthy individuals between 7 and 87 years of age. Cereb Cortex (2007) 17:1550–60. 10.1093/cercor/bhl066 16945978 PMC2329809

[18] Wilde EA Merkley TL Bigler ED Max JE Schmidt AT Ayoub KW. Longitudinal changes in cortical thickness in children after traumatic brain injury and their relation to behavioral regulation and emotional control. Int J Dev Neurosci. (2012) 30:267–76. 10.1016/j.ijdevneu.2012.01.003 22266409 PMC3322311

[19] Palacios EM Sala-Llonch R Junque C Fernandez-Espejo D Roig T Tormos JM. Long-term declarative memory deficits in diffuse TBI: correlations with cortical thickness, white matter integrity and hippocampal volume. Cortex (2013) 49:646–57. 10.1016/j.cortex.2012.02.011 22482692

[20] Michael AP Stout J Roskos PT Bolzenius J Gfeller J Mogul D. Evaluation of cortical thickness after traumatic brain injury in military veterans. J Neurotrauma (2015) 32:1751–8. 10.1089/neu.2015.3918 26131617

[21] Breslau N Chilcoat HD Kessler RC Davis GC. Previous exposure to trauma and PTSD effects of subsequent trauma: results from the Detroit Area Survey of Trauma. Am J Psychiatry (1999) 156:902–7. 10.1176/ajp.156.6.902 10360130

[22] Breslau N. Gender differences in trauma and posttraumatic stress disorder. J Gend Specif Med. (2002) 5:34–40. 11859685

[23] Nemeroff CB Bremner JD Foa EB Mayberg HS North CS Stein MB. Posttraumatic stress disorder: a state-of-the-science review. J Psychiatr Res. (2006) 40:1–21. 10.1016/j.jpsychires.2005.07.005 16242154

[24] Holm L Cassidy JD Carroll LJ Borg J Neurotrauma Task Force on Mild Traumatic Brain Injury of the WHO Collaborating Centre. Summary of the WHO collaborating centre for neurotrauma task force on mild traumatic brain injury. J Rehabil Med. (2005) 37**:**137–41. 10.1080/16501970510027321 16040469

[25] Caplan B MJE Edinburgh Handedness Inventory. New York, NY: Springer (2011).

[26] Kinnunen KM Greenwood R Powell JH Leech R Hawkins PC Bonnelle V. White matter damage and cognitive impairment after traumatic brain injury. Brain (2011) 134:449–63. 10.1093/brain/awq347 21193486 PMC3030764

[27] Fagerholm ED Hellyer PJ Scott G Leech R Sharp DJ. Disconnection of network hubs and cognitive impairment after traumatic brain injury. Brain (2015) 138:1696–709. 10.1093/brain/awv075 25808370 PMC4614120

[28] Arnett JA Labovitz SS Effect of physical layout in performance of the trail making test. Psychol Assess. (1995) 7:220–1. 10.1037/1040-3590.7.2.220

[29] Harman-Smith YE Mathias JL Bowden SC Rosenfeld JV Bigler ED. Wechsler Adult Intelligence Scale-Third Edition profiles and their relationship to self-reported outcome following traumatic brain injury. J Clin Exp Neuropsychol. (2013) 35:785–98. 10.1080/13803395.2013.824554 23947758

[30] Joy S Kaplan E Fein D. Speed and memory in the WAIS-III Digit Symbol–Coding subtest across the adult lifespan. Arch Clin Neuropsychol. (2004) 19:759–67. 10.1016/j.acn.2003.09.009 15288329

[31] Ruggiero KJ Del Ben K Scotti JR Rabalais AE. Psychometric properties of the PTSD Checklist-Civilian Version. J Trauma Stress (2003) 16:495–502. 10.1023/A:1025714729117 14584634

[32] King NS Crawford S Wenden FJ Moss NEG Wade DT. The rivermead post concussion symptoms questionnaire - a measure of symptoms commonly experienced after head-injury and its reliability. J Neurol. (1995) 242:587–92. 10.1007/BF00868811 8551320

[33] Fischl B Sereno MI Dale AM. Cortical surface-based analysis - II: inflation, flattening, and a surface-based coordinate system. Neuroimage (1999) 9:195–207. 10.1006/nimg.1998.0396 9931269

[34] Dickerson BC Fenstermacher E Salat DH Wolk DA Maguire RP Desikan R. Detection of cortical thickness correlates of cognitive performance: reliability across MRI scan sessions, scanners, and field strengths. Neuroimage (2008) 39:10–8. 10.1016/j.neuroimage.2007.08.042 17942325 PMC2141650

[35] Jovicich J Czanner S Han X Salat D van der Kouwe A Quinn B. MRI-derived measurements of human subcortical, ventricular and intracranial brain volumes: reliability effects of scan sessions, acquisition sequences, data analyses, scanner upgrade, scanner vendors and field strengths. Neuroimage (2009) 46:177–92. 10.1016/j.neuroimage.2009.02.010 19233293 PMC2866077

[36] Dale AM Fischl B Sereno MI Cortical surface-based analysis. I Segmentation and surface reconstruction. Neuroimage (1999) 9:179–94. 10.1006/nimg.1998.0395 9931268

[37] Fischl B Dale AM. Measuring the thickness of the human cerebral cortex from magnetic resonance images. Proc Natl Acad Sci USA. (2000) 97:11050–5. 10.1073/pnas.200033797 10984517 PMC27146

[38] Desikan RS Segonne F Fischl B Quinn BT Dickerson BC Blacker D. An automated labeling system for subdividing the human cerebral cortex on MRI scans into gyral based regions of interest. Neuroimage (2006) 31:968–80. 10.1016/j.neuroimage.2006.01.021 16530430

[39] Fischl B van der Kouwe A Destrieux C Halgren E Segonne F Salat DH. Automatically parcellating the human cerebral cortex. Cereb Cortex (2004) 14:11–22. 10.1093/cercor/bhg087 14654453

[40] Covassin T Elbin RJ. The female athlete: the role of gender in the assessment and management of sport-related concussion. Clin Sports Med. (2011) 30:125–31. 10.1016/j.csm.2010.08.001 21074087

[41] Colvin AC Mullen J Lovell MR West RV Collins MW Groh M. The role of concussion history and gender in recovery from soccer-related concussion. Am J Sports Med. (2009) 37:1699–704. 10.1177/0363546509332497 19460813

[42] Im K Lee JM Lee J Shin YW Kim IY Kwon JS. Gender difference analysis of cortical thickness in healthy young adults with surface-based methods. Neuroimage (2006) 31:31–8. 10.1016/j.neuroimage.2005.11.042 16426865

[43] Dum RP Strick PL. The origin of corticospinal projections from the premotor areas in the frontal lobe. J Neurosci. (1991) 11:667–89. 10.1523/JNEUROSCI.11-03-00667.1991 1705965 PMC6575356

[44] Kennis M Rademaker AR van Rooij SJ Kahn RS Geuze E. Resting state functional connectivity of the anterior cingulate cortex in veterans with and without post-traumatic stress disorder. Hum Brain Mapp. (2015) 36:99–109. 10.1002/hbm.22615 25137414 PMC6869264

[45] Lewen A Fredriksson A Li GL Olsson Y Hillered L. Behavioural and morphological outcome of mild cortical contusion trauma of the rat brain: influence of NMDA-receptor blockade. Acta Neurochir. (1999) 141:193–202. 10.1007/s007010050286 10189503

[46] Hermans EJ van Marle HJ Ossewaarde L Henckens MJ Qin S van Kesteren MT. Stress-related noradrenergic activity prompts large-scale neural network reconfiguration. Science (2011) 334:1151–3. 10.1126/science.1209603 22116887

